# Effect of Cinnamaldehyde and Citral Combination on Transcriptional Profile, Growth, Oxidative Damage and Patulin Biosynthesis of *Penicillium expansum*

**DOI:** 10.3389/fmicb.2018.00597

**Published:** 2018-03-29

**Authors:** Yuan Wang, Kewei Feng, Haihua Yang, Zhiwei Zhang, Yahong Yuan, Tianli Yue

**Affiliations:** ^1^College of Food Science and Engineering, Northwest University, Xi’an, China; ^2^College of Food Science and Engineering, Northwest A&F University, Yangling, China; ^3^Laboratory of Quality and Safety Risk Assessment for Agro-products (Yangling), Ministry of Agriculture, Beijing, China; ^4^National Engineering Research Center of Agriculture Integration Test (Yangling), Yangling, China; ^5^State Key Laboratory of Crop Stress Biology in Arid Areas, College of Agronomy, Northwest A&F University, Yangling, China; ^6^College of Food Science and Engineering, Qingdao Agricultural University, Qingdao, China

**Keywords:** cinnamaldehyde, citral, *P. expansum*, RNA-seq, PAT biosynthesis

## Abstract

*Penicillium expansum*, as a main postharvest pathogen of fruits, can secrete patulin (PAT), causing fruit decay and health problems. In this study, the antifungal test, SEM (scanning electron microscope) observation, transcriptional profile, PAT biosynthesis, and physiological characters of *P. expansum* exposed to cinnamaldehyde and citral combination (Cin/Cit) were evaluated. Cin/Cit could inhibit the mycelial growth and spore germination of *P. expansum* in a dose-dependent manner. Besides, Cin/Cit caused spores and mycelia wrinkled and depressed by SEM observation. Gene expression profiles of *P. expansum* were conducted by RNA sequencing (RNA-seq) in the presence or absence of Cin/Cit treatment. A total of 1713 differentially expressed genes (DEGs) were obtained, including 793 down-regulated and 920 up-regulated genes. Most of the DEGs participated in the biosynthesis of secondary metabolites, amino acid metabolism, and oxidation-reduction process, etc. Cin/Cit induced the dysfunction of the mitochondrial membrane, causing the potential influence on energy metabolism and reactive oxidative species production. The changes of superoxide dismutase (SOD) and catalase (CAT) activities combing with the increase of hydrogen peroxide content indicated the oxidative stress on *P. expansum* induced by Cin/Cit, which corresponded well with the transcriptional results. Moreover, both the RNA-seq data and the qRT-PCR showed the remarkable down-regulation of genes included in the PAT biosynthetic pathway under the Cin/Cit treatment. These findings provided more useful information about the antifungal mechanism of Cin/Cit against *P. expansum* at molecular and gene levels and suggested that Cin/Cit is a potential candidate to control *P. expansum*.

## Introduction

Fungal and mycotoxins contaminations are the major problems in agricultural products safety and human health. *Penicillium expansum* is one of the most devastating pathogens, which causes blue mold decay on many types of fruit. *P. expansum* can spread rapidly under the suitable condition and secretes PAT, a mycotoxin with potential mutagenic, carcinogenic, teratogenic and embryotoxic effects on humans (Moake et al., 2005). Currently, besides certain prophylactic measures applied to prevent the development of pathogen in the storage environments, the control of postharvest pathogens mainly depends on the synthetic fungicides (Lai et al., 2017). However, losses of the fungicides efficiency due to the emergence of fungicide-resistant pathogens, and public concern over chemical residues in food and environment have promoted investigations of alternative strategies to combat fungal decay and to increase food safety and public health (Tian et al., 2015).

Essential oils (EOs) are volatile liquid extracted from the natural raw material of plant, containing various individual constituents (ICs) such as terpenes, terpenoids, or aromatic and aliphatic constituents (de Souza et al., 2016). Most EOs and their ICs are cited as “generally recognized as safe” (GRAS) in food by the USFDA, and have proved to possess antimicrobial activities (Neri et al., 2006; Zhang et al., 2016). Cinnamaldehyde and citral are the major components of *Cinnamon bark* EO and *Cymbopogon citratus* EO, respectively, and have been reported to inhibit microbial growth as a botanical fungicide in fruit and its products. Cinnamaldehyde or cinnamaldehyde-emulsion was effectively applied to inhibit *Salmonella typhimurium* and *Staphylococcus aureus* in watermelon juice, *Escherichia coli* O157: H7 and *Salmonella enterica* in apple juice (Friedman et al., 2004; Jo et al., 2015). Additionally, the dual combination of the *trans*-cinnamaldehyde-citral emulsion at 100 μg/ml was proved to inhibit *Zygosaccharomyces bailii* in apple juice stored at 20°C for 27 days (Loeffler et al., 2014). Both cinnamaldehyde and citral exhibit direct inhibition to the pathogen, as well as indirectly control the pathogen growth by improving the host’s defense system (Fan et al., 2014; Jiang et al., 2015).

Researchers have investigated the complex action modes of EOs from various perspectives. The main mechanism is based on their lipophilic character, which facilitates the access of the hydrophobic compounds to the cytoplasmic membrane, causing membrane damage, loss of intracellular substances, and finally microbial death (de Souza et al., 2016). Cinnamaldehyde was reported to inhibit the growth of *Aspergillus flavus*, *Fusarium verticillioides*, *A. ochraceus*, and *E. coli*, *S. aureus* by cellular damage (Hua et al., 2014; Xing et al., 2014; Sun et al., 2016; Zhang et al., 2016). Citral exhibited its antimicrobial activity against *Cronobacter sakazakii*, *P. digitatum*, and *P. italicum* by affecting membrane function or integrity (Tao et al., 2014; Zheng et al., 2015; Shi et al., 2016). Although cinnamaldehyde or citral has been reported in control of *P. expansum*, the previous works were involved in antifungal activities and the application efficacy in food packaging (Neri et al., 2006; Xing et al., 2010; Camele et al., 2012; Balaguer et al., 2013; Manso et al., 2015). The knowledge of their action modes on *P. expansum* at the molecular level is rather limited, and thus requires further investigations.

Recently, RNA-seq technology has been used in many studies of gene expression, including the investigation of fungal response mechanism to EOs or ICs. Ouyang et al. (2016) demonstrated that citral inhibited *P. digitatum* by the down-regulation of ergosterol biosynthesis through transcriptional profiling analysis (Ouyang et al., 2016). In another report, the transcriptional profile of *Paracoccidioides lutzii* exposed to argentilactone, a constituent of the EO of *Hyptis ovalifolia*, was evaluated to investigate the response mechanism (Araujo et al., 2016). Additionally, RNA-seq technology has been used to analyze the molecular mechanism of fungi–host interaction, or fungal drug-resistance (Liu et al., 2015; Barad et al., 2016; Wang H. et al., 2016). Therefore, the present study is to evaluate the effect of Cin/Cit on growth, micromorphology, oxidative damage, and PAT biosynthesis of *P. expansum*. And the whole gene expression profile in *P. expansum* response to Cin/Cit was further analyzed based on the data from RNA-seq, in an effort to explore the molecular mechanism and key pathways or genes involved in.

## Materials and Methods

### Chemicals, Strain, and Growth Conditions

Analytical-grade cinnamaldehyde (95%) and citral (96%) were purchased from Jiangxi Xuesong Natural Medicinal Oil Co., Ltd., China. *P. expansum* F-WY-12-02 was isolated from dropped kiwifruits in our laboratory (Wang Y. et al., 2016) and were maintained in potato dextrose agar (PDA) and stored at 4°C. This isolate has previously shown to produce 146.58 μg/g of PAT in PDA plates after 10 days at 25°C. The minimum inhibitory concentration (MIC) was determined by dilution method in PDB (potato dextrose broth) medium as a previous work with minor modifications (Zhang et al., 2016). The EOs was dissolved in Tween 80 and added to the 10 ml sterile PDB medium to be saved as the stock solution. Then the EOs stock was diluted to obtain final concentrations ranging from 20 to 200 mg/L. Finally, 50 μl of 10^6^ spores/ml *P. expansum* conidia were added to each PDB medium (5 ml) containing different concentrations of EOs. Tubes were incubated at 25°C for 3 days under continuous shaking. Controls were performed in PDB medium with only the spore suspension. MIC was defined as the lowest concentration of EO with no visible fungal growth. The minimum fungicidal concentration (MFC) was measured by a subculture of 100 μl from each tube with no visible fungal growth on a PDA plate followed by incubation at 25°C for 3–5 days. MFC was defined as the lowest concentration of EO with initial inoculum fungi killed. Each test was performed in triplicate. The Fractional Inhibitory Concentration Index (FICI) of EOs was determined according to a previous study to explore the interaction of two EOs (Iten et al., 2009). The result showed that FICI of cinnamaldehyde and citral was 1, indicating their addition effect. Therefore, in the present study, a combined application of Cin/Cit was chosen against *P. expansum* F-WY-12-02. The MIC, MFC, and FICI of Cin/Cit were shown in Supplementary Table S1.

### Antifungal Effects of Cin/Cit on *P. expansum*

#### Spores Germination

Conidia from *P. expansum*, cultivated on PDA medium at 25°C for 7 days, was harvested using sterile distilled water containing 0.05% (v/v) Tween 80. Then 500 μl spore suspension (10^6^ spores/ml) were added to each sterile tube containing 1.5 ml Cin/Cit solution, providing the final concentrations of 1/4 MIC, 1/2 MIC, MIC, 2 MIC. Tubes were incubated at 25°C for 12 h with 120 rpm. At the end of the incubation period, germinated spores were observed using a light microscope (Olympus, Tokyo, Japan). Germination was defined as the length of the germ tube was greater than or equal to the diameter of the swollen spore. The percentage of germinated spores was calculated as *P*(%) = (*N*_germinated spores_/*N*_total spores_) 100%.

#### Mycelial Growth

Influence of Cin/Cit on the growth of *P. expansum* was evaluated by colony diameter and mycelial dry weight. For colony diameter, 50 μl of 10^6^ spores/ml was spotted on the center of PDA plate containing 1/4 MIC-2 MIC of Cin/Cit. Plates were sealed using Parafilm and incubated for 120 h at 25°C. Colony diameter was measured every 24 h using the cross method. PDA plates without Cin/Cit were set as control. The radial growth inhibition after 120 h was evaluated. The inhibitory effect of Cin/Cit on the mycelial weight was determined as described previously (Sun et al., 2016). The concentration of Cin/Cit ranged from 1/4 MIC-2 MIC. Incubation was conducted at 25°C for 120 h with agitation (120 rpm). After filtering and washing, the inhibition of mycelial weight at the end of incubation was measured. Three replicates were performed for each treatment. Growth inhibition was calculated by the equation: *I* (%) = (*D*c (*W*c) -*D*t (*W*t)) × 100/*D*c (*W*c). I: inhibition of mycelial growth; *D*c (*W*c): colony diameter or mycelial weight in control group; *D*t (*W*t): colony diameter, or mycelial weight in the treatment group.

### Scanning Electron Microscopy (SEM) Analysis

Mycelia from 72 h incubation of *P. expansum* in PDB were collected and washed with PBS (pH 7.4). Then 0.5 g of wet mycelia were added in tubes containing 5 ml PDB and Cin/Cit at 0, MIC, 2 MIC. For spores, 100 μl of Cin/Cit was added to tubes containing 4.9 ml of spores suspension (10^7^ spores/ml), reaching the final concentration of 0, MIC, 2 MIC. After incubation at 25°C, 120 rpm for 12 h, both mycelia and spores were harvested by centrifugation for 5 min at 10, 000 × *g* and washed twice with PBS, followed by a series of pretreatments based on Sun et al. (2016). At last, the micromorphology was observed using a scanning electron microscope (S-3400N, Hitachi, Tokyo, Japan).

### Transcriptome Analysis

Spores of *P. expansum* were inoculated in PDB medium supplemented with 0 or 1/2 MIC of Cin/Cit and cultured at 25°C, 120 rpm for 5 days. Each group has two biological replicates. The harvested mycelia were quickly frozen with liquid nitrogen for total RNAs preparation, RNA quality detection, cDNA libraries construction and RNA-seq, which was performed at Novogene Bioinformatics Technology Co., Ltd. (Beijing, China). Total RNA was extracted using TRIzol reagent (Invitrogen, United States) according to the manufacturer’s instruction and treated with RNase-free DNase I. The agarose gel electrophoresis, a NanoDrop^®^2000 spectrophotometer (Thermo Scientific, Wilmington, DE, United States), a Qubit^®^Fluorometer 2.0 (Life Technologies, Carlsbad, CA, United States) and an Agilent 2100 bioanalyzer (Agilent Technologies, Santa Clara, CA, United States) were used to test the concentration and integrity of RNA samples (Wang H. et al., 2016). The cDNA libraries were constructed according to previous reports and were sequenced on the Illumina HiSeq2000 platform (HiSeq2000, Illumina, San Diego, CA, United States, 2010) (Lin et al., 2013; Lai et al., 2017). The resulting RNA-seq reads were mapped onto the reference genome of *P. expansum* Link isolate d1 from Israel (denoted as PEXP) using Tophat v2.0.12 (Kim et al., 2013; Ballester et al., 2015).

Additionally, the followed analysis, including quantification of gene expression (HTSeq v0.6.1), identification of differentially expressed genes (DEGs) (DESeq v1.10.1), enrichment analysis of DEGs by GO enrichment analysis (GOSeq Release2.12) and KEGG pathways analysis (KOBAS v2.0), were carried out based on a previous report (Wang H. et al., 2016). The RNA-Seq data have been deposited in the Genome Sequence Archive in BIG Data Center, Beijing Institute of Genomics (BIG), Chinese Academy of Sciences, with accession code CRA000582.

### Mitochondrial Membrane Potential (MMP) Measurement

The effect of Cin/Cit on MMP of *P. expansum* was evaluated using 1 μg/ml of rhodamine 123 (RH123, Sigma-Aldrich), respectively. A conidial suspension (10^7^ spores/ml) was prepared in PBS with 2% (w/v) D-glucose. The cells were incubated with various concentrations of Cin/Cit (0, 1/2 MIC, 2 MIC) at 25°C, 120 rpm for 10 h (Tian et al., 2015). Finally, the spores were collected, washed with PBS and stained for 15 min at room temperature in the dark. After that, the spores were washed twice with PBS and then observed by flow cytometer (BD Biosciences, United States) with the excitation, and emission wavelength as 488 and 525 nm, respectively. Unstained cell suspensions were included as autofluorescence controls. Each treatment included three replicates.

### Determination of Glucan, Chitin, and Protein of Cell Wall

After Cin/Cit treatment (0, 1/2 MIC) for 5 days, mycelia were filtered and washed with PBS, then cell walls were isolated and analyzed using the acid (H_2_SO_4_) treatment method (Francois, 2006). Cell wall monosaccharide concentrations were normalized using the total dry cell wall mass for individual samples. Hot alkaline (NaOH) method was used to extract cell wall proteins. The amounts of proteins in cell wall extracts were measured by Bradford method (Bradford, 1976) using bovine serum albumin as a standard.

### Hydrogen Peroxide (H_2_O_2_) Measurement

Mycelia from 5 days incubation in PDB in presence or absence of Cin/Cit (0, 1/2 MIC) were collected by filtration and washed with PBS. After that, the mycelia were subjected to the determination of H_2_O_2_ based on the previous method (Jiang et al., 2015). The H_2_O_2_ content was calculated using H_2_O_2_ as a standard and the result was expressed as μmol/g mycelia.

### Assay of SOD and CAT Activity

Mycelia were homogenized in cold PBS (10 mM, pH 7.0) by quartz sand. After that, the homogenates were centrifuged at 13, 000 × *g* for 30 min at 4°C. The proteins and enzymes were analyzed in the supernatants (Sun et al., 2016). The determination of proteins contents in extract fluids was based on Bradford’s method (Bradford, 1976). superoxide dismutase (SOD) activity was assayed by its ability to inhibit the photochemical reduction of nitrotetrazolium blue chloride (NBT) at 560 nm (Beauchamp and Fridovich, 1971). The SOD activity was defined as units per milligrams of protein (U/mg pro). CAT activity was determined according to a previous method (Candan and Tarhan, 2003). The specific CAT activity was expressed as U/mg protein/min.

### Detection of PAT Production and Related-Genes Expression

Spores of *P. expansum* were cultured statically in PDB medium (10^5^ spores/ml) supplemented with 0, 1/2 MIC, MIC of Cin/Cit at 25°C, 120 rpm for 5 days. The culture solutions were filtered to separate mycelium and filtrate. Mycelia were washed, dried and weighted. The filtrate was collected for PAT determination. PAT was extracted with ethyl acetate by AOAC Official Method 2000.02 (MacDonald et al., 2000). Analysis of PAT was performed on the HPLC (LC-2010A; Shimadzu, Kyoto, Japan) system. The analytical column used was Eclipse Plus C18 (250 mm × 4.6 mm i.d., 5 mm, Agilent Technologies, United States). The eluent used was acetonitrile-water (10: 90, v/v) at a flow rate of 1.0 ml/min and the sample injecting volume was 20 μl. The UV detector wavelength was set at 276 nm.

Under 0 or 1/2 MIC Cin/Cit stress, conidia of *P. expansum* were cultured in PDB medium at 25°C, 120 rpm for 5 days. After that, mycelia were harvested and subsequently flash-frozen with liquid nitrogen, ground to fine powder, and stored at -80°C for RNA isolation. The total RNA was isolated using the Fungal RNA Kit (Omega Bio-Tek, Inc. Norcross, Ga., United States) according to the manufacturer’s instructions. Genomic DNA elimination and first strand cDNA synthesis were carried out by PrimeScript RT reagent Kit with gDNA Eraser (Takara Bio Inc., Japan) according to the protocol of producer. The qRT-PCR (quantitative real-time polymerase chain reaction) was performed using SYBR *Premix Ex Taq*^TM^ II (Takara Bio Inc., Japan) in a Thermal Cycler Dice^TM^ Real-Time System (Thermal, United States). The primer pairs for the specific 15 genes involved in PAT biosynthesis were synthesized based on a previous study (Zong et al., 2015). The PCR conditions were as follows: 95°C for 30 s, followed by 40 cycles of 95°C for 5 s, 60°C for 30 s. The change in fluorescence of SYBR Green in every cycle was monitored by the system software, and the threshold cycle (*C*t) over the background was calculated for each reaction. The sample was normalized using β-tubulin and the relative expression levels were measured using the 2^(-ΔΔC_t_)^ analysis method.

### Statistical Analysis

Except for the transcriptome analysis, all the data were expressed as the mean ± SD by measuring three independent replicates and were performed using SPSS version 22.0 (SPSS Inc., IBM, Armonk, NY, United States) and Origin 9.0 (Origin Lab Corporation, United States). Significance was evaluated by one-way analysis of variance (ANOVA). Tukey’s multiple-range test was performed following a significant (^∗^*p* < 0.05, ^∗∗^*p* < 0.01) test.

## Results and Discussion

### Antifungal Effects of Cin/Cit on *P. expansum* F-WY-12-02

**FIGURE 1:**
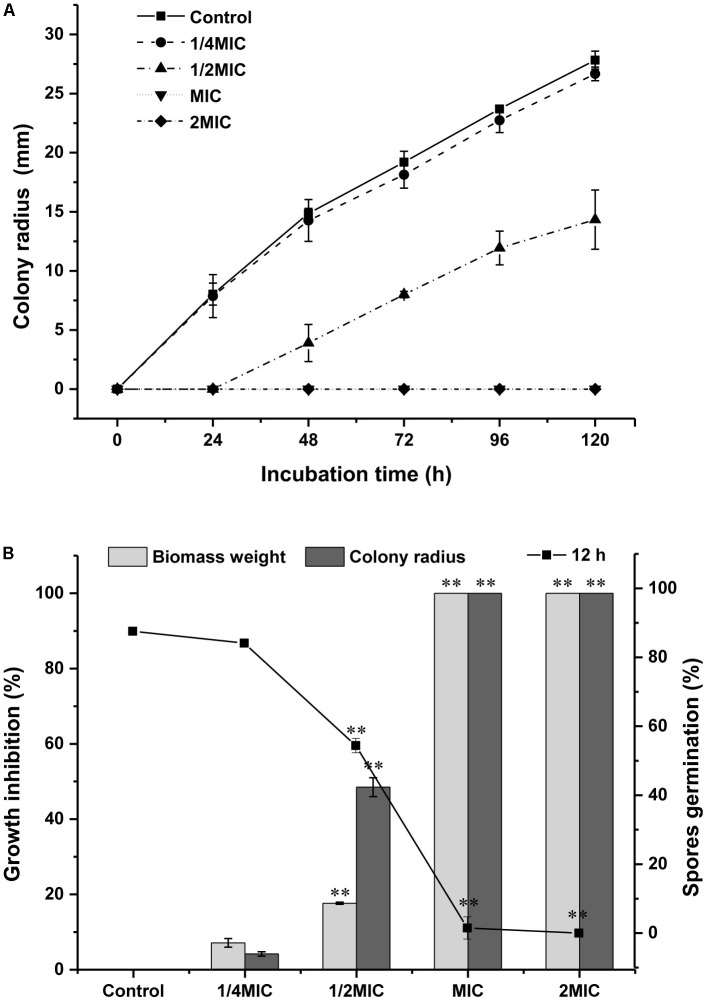
Effect of Cin/Cit on the mycelial growth and spore germination of *Penicillium expansum* F-WY-12-02. **(A)** Colony radius. **(B)** Spore germination (%) at 12 h, growth inhibition (%) for biomass weight and colony radius. ^∗∗^*p* < 0.01.

Inhibitory effects of Cin/Cit on the colony growth, biomass, and spore production of *P. expansum* were presented in **Figure 1**. Cin/Cit exhibited the capacity to delay or inhibit fungal growth in a dose-dependent manner. The extension of the colony radius and the accumulation of the biomass weight of *P. expansum* were completely inhibited by Cin/Cit at MIC and 2 MIC during the whole incubation period (**Figures 1A,B**). After 5 days incubation, the lowest concentration of Cin/Cit (1/4 MIC) inhibited the extension of colony radius and the increase in biomass weight by 4.19 and 7.12%, respectively. With regard to the spore germination rate of *P. expansum* at 12 h, spores treated with 1/4 MIC (84.1%) showed no significant difference compared with controls (87.6%). Additionally, there were no conidia formed in PDA plates filled with MIC and 2 MIC of Cin/Cit. Overall, it is obvious that Cin/Cit could slow down or totally inhibit the colony extension, mycelia biomass accumulation and spore germination of *P. expansum*. The inhibitory effects are related to the dose and treatment duration.

### SEM Observation

**FIGURE 2:**
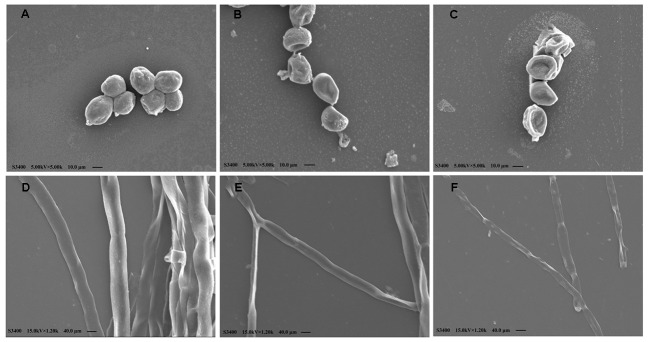
Scanning electron microphotographs of *P. expansum* F-WY-12-02. **(A)** Untreated spores. **(B)** Cells treated with Cin/Cit at 1/2 MIC. **(C)** Cells treated with Cin/Cit at 2MIC. **(D)** Untreated mycelia. **(E)** Mycelia treated with Cin/Cit at 1/2 MIC. **(F)** Mycelia treated with Cin/Cit at 2 MIC.

To understand the action mode of Cin/Cit against *P. expansum*, we started with evaluating the morphological alteration in *P. expansum* spores and mycelia by SEM (**Figure 2**). There were significant morphological differences between controls and treated *P. expansum* cells. The controls appeared to be normal with intact and plump spheroidal or tubular structure. After exposure to Cin/Cit at MIC, the spores and hyphae became wrinkled, thinner and exhibited clear depressions on the surface. The degrees of morphological modifications increased as Cin/Cit concentration increased. The morphological changes of *P. expansum* under Cin/Cit treatment were similar to some fungi and bacteria, such as *A. flavus*, *F. verticillioides*, *E. coli*, and *S. aureus* treated by cinnamon essential oil (Xing et al., 2014; Sun et al., 2016; Zhang et al., 2016). The wrinkled and thinner surface of spores or mycelia may be due to the release of cellular content.

### Overall Transcriptional Response Profile of *P. expansum* to Cin/Cit

To explore the potential inhibitory mechanism of Cin/Cit on *P. expansum*, a transcriptome analysis was carried out to assess the specific response at mRNA levels. The details in assembly and annotation information were shown in **Table 1**. Via RNA-seq, averagely 30.69 million and 31.42 million raw reads were generated from control and treatment samples, respectively. After filtering the adaptor sequences, 29.13 million and 29.73 million clean reads were obtained. Therein, 74.15 and 73.28% of total clean reads from control and Cin/Cit group were aligned to reference sequences. A total of 1713 genes were considered as significant changes in abundance under Cin/Cit stress. Among them, the expressions of 793 DEGs were down-regulated and of 920 DEGs were up-regulated.

**Table 1:** Summary of RNA-seq reads in control (PeC) and treatment (PeM) groups of *Penicillium expansum* F-WY-12-02.

Parameter	PeC	PeM	PeC and PeM
Raw reads	30694374	31424213	
Clean reads	29133442	29730617	
Total mapped	74.15%	73.28%	
Clean bases	4.37 G	4.46 G	
Error rate (%)	0.02	0.02	
Q20 (%)	95.45	95.81	
Q30 (%)	90.04	89.71	
GC content (%)	52.07	52.48	
The number of all genes			11468
Genes annotation against GO			8055
DEGs annotation against GO			1261
Genes annotation against KEGG			4029
DEGs annotation against KEGG			842
Up-regulated genes		920	
Down-regulated genes		793	

### Functional Classification and Pathway Analysis of DEGs

**FIGURE 3:**
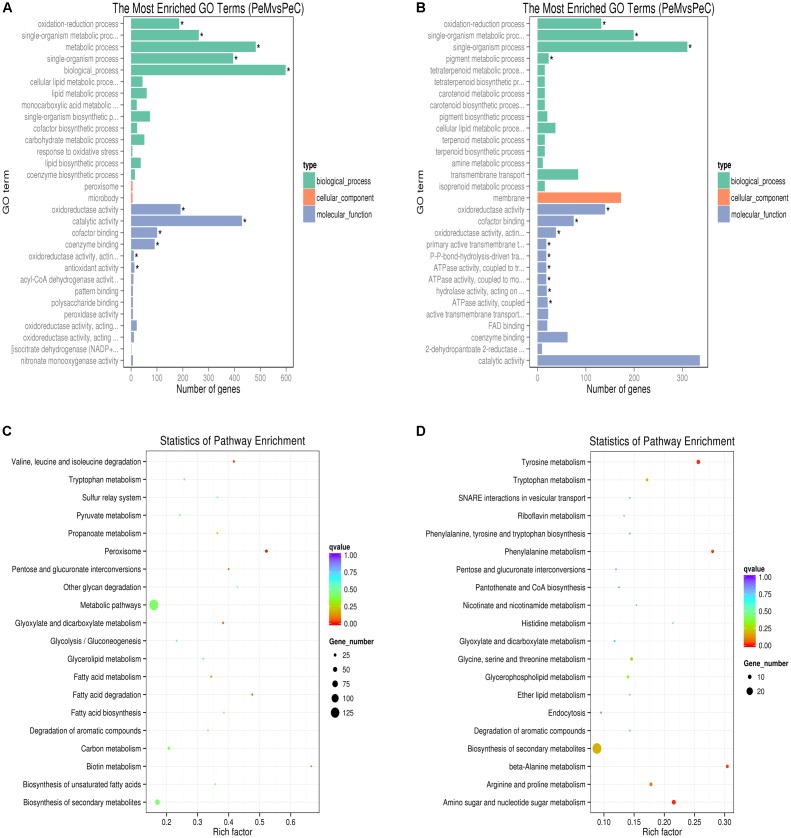
GO functional classification **(A,B)** and KEGG enrichment **(C,D)** of up-regulated **(A,C)** and down-regulated **(B,D)** DEGs. **(A,B)** The ordinate means GO term, the abscissa means the number of DEGs of each GO term. Different color means biological process, cellular component, and molecular function, respectively. “^∗^” means significant enrichment. **(C,D)** The ordinate means the name of the pathway, the abscissa means rich factor. The size of the plot means the number of DEGs in one KEGG, the color of the plot close to red means more significant enrichment.

The DEGs between the two libraries (PeC and PeM) provided a clue to the molecular mechanism related to the *P. expansum* response to Cin/Cit. Genes with different expression were related to a wide variety of regulatory and metabolic processes. To better analyze the functions, metabolic pathways and interactions of the 1713 DEGs, GO, and KEGG enrichment analyses were performed. A total of 1261 DEGs were mapped to 2289 GO terms. Among of which, 1361, 658, and 270 GO terms belonged to biological process, molecular function and cellular component, respectively. **Figure 3A** showed the top 30 enriched functional categories of 920 up-regulated DEGs. Therein, oxidation-reduction process, single-organism metabolic process, and metabolic process, etc. were significant enrichment terms in the biological process. Oxidoreductase activity, catalytic activity, cofactor binding, etc. were the main functional terms in molecular function. For the down-regulated DEGs (**Figure 3B**), oxidation-reduction process, single-organism metabolic process, and pigment metabolic process, etc. were the most abundant belonging to the biological process. Additionally, most of the significant enrichment terms in the molecular function were related to oxidoreductase activity, ATPase activity, transmembrane transporter activity, etc. DEGs were mapped to 90 KEGG pathways. Therein, the most abundant DEGs (171) were enriched in the metabolic pathway (pcs01100), 85 DEGs were enriched in Biosynthesis of secondary metabolites (pcs01110), 28 and 27 DEGs were enriched in Biosynthesis of amino acids (pcs01230), and Carbon metabolism (pcs01200), respectively. Moreover, the pathways affected in the present work may be related to two processes, including spores germination and mycelial growth. **Figures 3C,D** showed the scatter plots of the top 20 KEGG enrichment of up-regulated and down-regulated DEGs, respectively. The KEGG enrichment results showed that the pathways which were sensitive to the Cin/Cit stress mainly belonged to the peroxisome, amino acids metabolism or degradation, fatty acid metabolism, biosynthesis or degradation, biosynthesis of secondary metabolites etc. Those sensitive pathways associated with genetic information, energy metabolism, cell integrity, cell membrane, oxidation-reduction process, etc. will be further analyzed.

### Genes Involved in Stress Response

To alleviate the unfavorable growth situations caused by Cin/Cit treatment, *P. expansum* cells will develop relative responses by adjusting gene expression pattern. In the present study, considerable alterations in gene expression levels related to stress response were found in *P. expansum*. Genes associated with ribosome biogenesis, RNA degradation, RNA transport and mRNA surveillance pathway were partially depressed, suggesting that a negative influence in the translational activity of cells. A similar result was observed in *P. digitatum* cells treated with citral (Ouyang et al., 2016).

**FIGURE 4:**
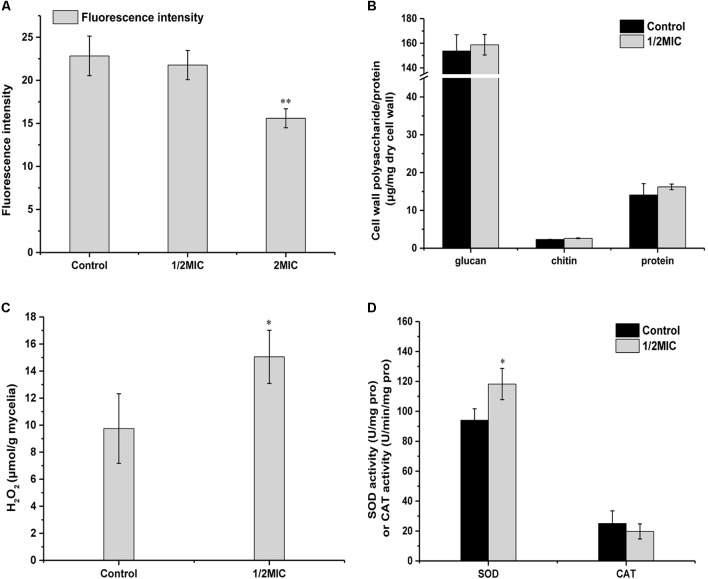
Effect of Cin/Cit on MMP **(A)**, cell wall polysaccharide and protein **(B)**, H_2_O_2_ content **(C)**, SOD and CAT activities **(D)** of *P. expansum* F-WY-12-02. *Bars* represent the standard deviation of the treatment means. ^∗^*p* < 0.05, ^∗∗^*p* < 0.01.

The repressions in most genes of energy-related pathways including nitrogen metabolism, oxidative phosphorylation, glycolysis/gluconeogenesis, etc. were observed in the present work. For instance, the gene (PEXP_101190) relative to aldehyde dehydrogenase in glycolysis pathway was down-regulated by 2.43-fold. Additionally, mitochondria play an important role in producing energy (ATP) through oxidative phosphorylation to regulate cellular metabolism (Heazlewood et al., 2004). In the present work, the dysfunction of mitochondrial membrane potential (MMP) has been observed using RH123 by flow cytometer (**Figure 4A**). The fluorescent intensity of *P. expansum* cells decreased significantly (*p* < 0.05) after Cin/Cit exposure at 2 MIC (15.59), compared with controls (22.83) and 1/2 MIC (21.77), suggesting the loss or depolarization of MMP and hence likely of their function (Araujo et al., 2016). Some inhibitors of mitochondrial electron transport could reduce MMP, leading to the reduction of ATP production and cell death (Wu et al., 2009). Therefore, the disturbance of MMP indirectly supported the down-regulation of the DEGs involved in the energy-metabolism process. Citral was reported to alter the mitochondrial morphology, reduce ATP content and inhibit the TCA cycle of *P. digitatum* (Zheng et al., 2015). The down-regulation of most genes related to energy-related pathways may be attributable to two aspects. On the one hand, Cin/Cit may cause cellular dysfunction, leading to an inhibitory effect on respiration and energy metabolism; on the other hand, the reduction in energy metabolism may be a feedback or response to the unfavorable environmental conditions to keep the cell viability. In the present work, some genes related to heat shock protein, an important molecular marker of the stress response, were up-regulated from 1.86- to 2.28-fold, such as *hsp 90* and *hsp 70*. Researchers have suggested that *hsp 90* is a molecular chaperone that plays important roles in the regulation of several signaling systems, and is required for viability under stress conditions in eukaryotes (Thomas and Baneyx, 1998; Ali et al., 2006). A previous study reported that genes of *Paracoccidioides* spp. responding to stress, including *hsp 90* were up-regulated after argentilactone treatment (Araujo et al., 2016).

### Genes Related to Cell Integrity

#### Cell Wall

The cell wall is the outermost barrier of fungi against the environmental pressure and is essential for fungal survival. In the present work, some DEGs related to cell wall biosynthesis, including amino sugar and nucleotide sugar metabolism, starch and sucrose metabolism, were influenced by Cin/Cit. Of the 17 DEGs related to chitin synthase, chitinase, glycoside hydrolase, mannose-6-phosphate isomerase etc. in amino sugar and nucleotide sugar metabolism, 11 DEGs were down-regulated from 1.45- to 4.7-fold, and the other six DEGs were up-regulated from 1.07- to 2.38-fold. Additionally, all of the 12 DEGs belonging to starch and sucrose metabolism were up-regulated by 1.54 to 8.81-fold, among which seven DEGs were related to glycoside hydrolase, two DEGs encoded for pectinesterase and α-amylase, respectively. The genes above were all involved in the formation of cell wall architecture. Chitinase, an important degradation enzyme, is conducive to fungal cell separation during their reproduction period. Therefore, the down-regulation of DEGs encoding for chitinase may indirectly influence the fungal cell separation of *P. expansum*, causing fungal reproduction disorder. In the present work, we analyzed the changes of cell wall glucan, chitin and protein of *P. expansum* exposed to Cin/Cit (**Figure 4B**). Glucan was the predominant components in both control and treated cell wall skeleton with the content 153.76 and 158.76 μg/mg mycelia, respectively. Chitin content of the cell wall in the presence or absence of Cin/Cit treatment was 2.61 and 2.32 μg/mg mycelia, respectively. Although the content of glucan and chitin slightly increased after Cin/Cit treatment, no significant differences were found compared to the controls (*p* > 0.05). Additionally, the variation trend of cell wall proteins before and after Cin/Cit treatment (14.09–16.21 μg/mg mycelia) was similar to the cell wall polysaccharides. The slight increases in cell wall polysaccharides and proteins were probably due to the defensive response or the compensatory mechanism of cells to overcome the external stress (Parveen et al., 2004). The previous study has reported the similar phenomenon that limonene treatment caused several genes involved in cell wall integrity signaling pathway over-expressed, while no significant changes in the cell wall polysaccharide compositions of *Saccharomyces cerevisiae* (Brennan et al., 2013).

#### Cell Membrane

Considering the lipophilic character of EOs, the microbial cytoplasmic membranes have been regarded as the target of these bioactive compounds (da Cruz Cabral et al., 2013). RNA-seq data showed that the expression levels of some genes involved in cell membrane compositions or function-related pathways were influenced after Cin/Cit treatment, such as fatty acids (FAs) biosynthesis, biosynthesis of unsaturated FAs, FAs metabolism, FAs degradation, steroid biosynthesis, etc. Five DEGs in the biosynthesis of unsaturated FAs pathway were all up-regulated by 1.66- to 4.59-fold. Of the 12 DEGs related to FAs metabolism pathway, 11 DEGs were induced over-expressed from 1.22- to 4.59-fold. There were 12 DEGs related to FAs degradation pathway, among which 10 DEGs were up-regulated from 1.05- to 4.99-fold. The above results indicated that Cin/Cit probably affected the membrane fluidity or permeability by intervening FAs compositions and contents. Researchers have revealed that microbial cells will respond to the environmental stresses by modulating the ratio of saturated to unsaturated FAs, *cis* to *trans* unsaturation. And the unsaturated FAs play an important role in responding to various external stresses (Wu et al., 2012).

Ergosterol is one of the major sterol components in the fungal membrane and is considered crucial to regulate cell structure, osmosis, growth, and proliferation (Ouyang et al., 2016). In the present work, the expression level of one DEG (PEXP _052220, *ERG6*) encoding ergosterol biosynthesis methyltransferase in ergosterol biosynthetic pathway, was down-regulated by 1.51-fold. This enzyme catalyzes the zymosterol into fecosterol, an intermediate of ergosterol biosynthesis. A previous study showed that *ERG6* gene deletion caused *Candida albicans* cell lose the ability to synthesize ergosterol (Jensenpergakes et al., 1998).

In the present study, the most down-regulated DEGs were enriched in the biological process and molecular function (**Figure 3B**). However, the only one GO term related to the cellular component belonged to membrane function containing 173 DEGs. Therefore, based on the analysis of the expression levels and functions of above genes, membrane structure may be the main target of Cin/Cit.

### Genes Involved in Redox System

In this work, the functional analysis of DEGs indicated that five DEGs related to the response to oxidative stress were all up-regulated from 1.47- to 3.78-fold. An increase of 1.54-fold in the expression of a gene related to SOD was also observed. Besides, some DEGs involved in peroxidase were up-regulated from 1.47- to 3.77-fold, such as cytochrome C peroxidase protein (CCP) which catalyzed the reduction of H_2_O_2_ using cytochrome C as an electron donor (Araujo et al., 2016). Previous studies have revealed that cellular oxidative stress could result in an overexpression of SOD and CCP in *S. cerevisiae* and *Paracoccidioides* spp. (Kwon et al., 2003; Araujo et al., 2016). Therefore, Cin/Cit seemed to stimulate oxidative stress in *P. expansum* cells, and the fungus represented an antioxidant defense system to prevent cell damage by inducing antioxidant enzyme.

Reactive oxygen species (ROS) production is commonly caused by some cellular stresses. H_2_O_2_ is often regarded as one of the main compounds of ROS as it can lead to the production of more reactive species, cause oxidative stress and decrease the viability of cells (Chen et al., 2015). As shown in **Figure 4C**, the content of H_2_O_2_ was significantly (*p* < 0.05) enhanced by 54.37% in mycelia treated with Cin/Cit, compared with controls, confirming that Cin/Cit caused oxidative stress in *P. expansum* cells. Antioxidant enzymes SOD and CAT, play a crucial role to protect cells against ROS. In this study, CAT activity in treated mycelia was reduced by 21.08% compared with the controls (**Figure 4D**). Conversely, SOD activity in Cin/Cit treatment was significantly enhanced by 25.67%, corroborating the transcriptional data. The opposite changes of the activity of CAT and SOD were similar to a previous research conducted in *A. flavus* (Sun et al., 2016). As shown in Supplementary Figure S1, the activity alterations of CAT and SOD were closely related to the simultaneous increase in the H_2_O_2_ content, which explained the increased level of H_2_O_2_ from the enzymology view. Mitochondria are widely known as a major source of ROS. The dysfunction of the MMP led to an influence on electron transport and changes in the proton gradient, which would cause the production of ROS and cellular oxidative stress (**Figure 4A**) (Jezek and Hlavatá, 2005; Suski et al., 2012). These results combining with the transcriptional data confirmed the hypothesis that Cin/Cit could act on mitochondria, and induce the oxidative stress in *P. expansum* cells.

### Gene Associated With ABC Transporter System

ABC (ATP-Binding Cassette) transporters energize diverse biological systems by using the hydrolysis of ATP, such as the export or import of various substrates, particularly the essential nutrients to microbial cells. Ten DEGs involved in ABC transporters were down-regulated from 1.03- to 3.45-fold, and two DEGs related to ATPase activity were repressed by 2.25-fold. Notably, the expression of one DEG (PEXP_101560) associated with amino acid/polyamine transporter I was decreased by 7.71-fold, indicating that Cin/Cit probably have great impacts on amino acids (AAs) transporter activity. The dysfunction of AAs transporter can limit the transport and consumption of AAs, and influence the protein biosynthesis, which will block some AAs metabolism-related pathways (**Figure 3D**) and physiological functions in fungal cells. ABC transporter system was also associated with fungal multidrug resistance, such as CDR (candida drug resistance) ABC transporter, multidrug resistance protein (MRP)/multi-antimicrobial extrusion protein, drug/metabolite transporter, etc. Four DEGs (PEXP_105370, PEXP_024900, PEXP_034130, PEXP_086290) belonging to the multidrug resistance protein, were down-regulated by 3.07, 2.81, 1.51, and 4.10-fold, respectively. Additionally, the DEG associated with drug/metabolite transporter (PEXP_068020) was down-regulated by 1.2-fold. Four DEGs involved in CDR ABC transporter were repressed from 2.26- to 4.84-fold. CDR ABC transporter is closely related to the drug efflux mechanisms, and the over-expression of its relative genes probably cause the multidrug resistance in fungal cells. Ouyang et al. (2016) have demonstrated that citral impaired the multidrug resistance in *P. digitatum* cell by decreasing the expression levels of multidrug resistance-associated protein genes. Therefore, based on our RNA-seq results, Cin/Cit probably reduced the drug resistance development in *P. expansum*.

### Cin/Cit Interferes With PAT Synthesis and Expression of Genes Involved in PAT Biosynthesis

The complete PAT biosynthesis pathway, encoded by a 15-gene cluster named *PatA* to *PatO* has been identified in *P. expansum* (Li et al., 2015). Among the 15 genes in *P. expansum*, nine encode biosynthetic enzymes, three encode transporters (*PatA, PatC*, and *PatM*), one encodes a putative transcription factor (*PatL*), and two (*PatF* and *PatJ*) have an unknown function.

**FIGURE 5:**
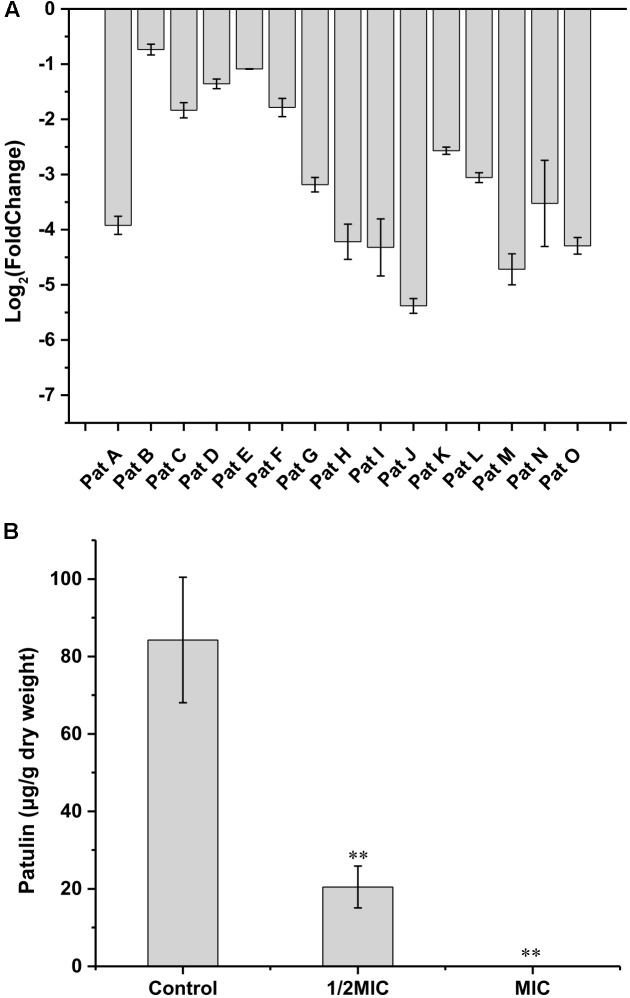
Effect of Cin/Cit on expressions of 15 genes involved in PAT biosynthetic pathway **(A)**, and PAT biosynthesis **(B)** of *P. expansum* F-WY-12-02. ^∗∗^*p* < 0.01.

Based on the transcriptome data, the putative function, and expression level of each gene in PAT gene cluster were listed in **Table 2**. Therein, three genes (*PatB*, *PatG*, and *PatL)* showed no significant differences, while the other 12 genes were significantly down-regulated when *P. expansum* was treated with Cin/Cit. The *PatK* gene, encoding 6-methylsalicylic acid synthase (*6-MSAS*) which catalyzed the first step of PAT biosynthesis, was down-regulated by 6.77-fold. The down-regulation of *PatK* probably caused a reduction of the biosynthesis of 6-methylsalicylic acid as a main precursor of PAT. Another remarkable decrease by 6.07-fold was the expression of *PatJ*. Although the exact function of *PatJ* (Hypothetical protein II) was unclear, Zong et al. (2015) found that the mutant strain PeΔ*PatJ* presented lower growth rate and lost the ability to secrete PAT, speculating that *PatJ* might be involved in the sixth step of PAT pathway. The *PatA*, *PatC*, and *PatM* genes encoded acetate transporter, MFS (major facilitator superfamily) transporter, and ABC transporter, respectively. Of the three genes, *PatM* was repressed by 4.83-fold, indicating that ABC transporter may be more susceptible to the influence of external adverse environment among the cellular transporters. Additionally, the *PatH*, *PatI*, *PatN*, and *PatO* genes were down-regulated by 3.82, 1.79, 3.525, and 3.525-fold, respectively. *PatH* and *PatI*, involved in the third and fourth steps of PAT biosynthesis pathway, respectively (White et al., 2006; Artigot et al., 2009). *PatN* gene encoded isoepoxydon dehydrogenase that catalyzed the conversion of isoepoxydon to phyllostine. A previous study showed that the deletion of the *PatN* gene in a *P. expansum* strain resulted in an 87.5% decrease in PAT production (Barad et al., 2013). According to the RNA-seq results in this study, Cin/Cit created a PAT restrictive condition to *P. expansum*, so that the expressions of most genes involved in PAT biosynthetic pathway, particularly the early steps, were significantly down-regulated, which were similar to the previous observations (Tannous et al., 2014; Lai et al., 2017). Furthermore, the qRT-PCR was performed and demonstrated that the expressions of all the 15 genes were down-regulated under Cin/Cit stress (1/2 MIC) (**Figure 5A**). Meanwhile, PAT production was also significantly (*p* < 0.01) decreased by 75.69% and 100% in Cin/Cit group at 1/2 MIC and MIC, respectively (**Figure 5B**).

**Table 2:** Function and expression of the 15 genes in PAT biosynthetic pathway of *P. expansum* F-WY-12-02 after Cin/Cit treatment.

Gene	Gene_id	Putative function	Gene expression	Log_2_ Fold Change
*PatA*	PEXP_094390	Acetate transporter	DOWN	-3.7148
*PatB*	PEXP_094380	Carboxylesterase	FALSE	-0.83452
*PatC*	PEXP_094370	MFS transporter	DOWN	-2.132
*PatD*	PEXP_094360	Alcohol dehydrogenase	DOWN	-1.6216
*PatE*	PEXP_094350	GMC oxidoreductase	DOWN	-1.1296
*PatF*	PEXP_094340	Hypothetical protein I	DOWN	-2.1206
*PatG*	PEXP_094330	Amidohydrolase family protein	FALSE	-1.3793
*PatH*	PEXP_094320	m-Cresol methyl hydroxylase	DOWN	-3.8206
*PatI*	PEXP_094440	m-Hydroxybenzyl alcohol hydroxylase	DOWN	-1.7929
*PatJ*	PEXP_094450	Hypothetical protein II	DOWN	-6.0695
*PatK*	PEXP_094460	6-Methyl salicylic acid synthase	DOWN	-6.7733
*PatL*	PEXP_094430	C6 transcription factor	FALSE	0.39025
*PatM*	PEXP_094400	ABC transporter	DOWN	-4.8353
*PatN*	PEXP_094410	Isoepoxydon dehydrogenase	DOWN	-3.5252
*PatO*	PEXP_094420	Isoamyl alcohol oxidase	DOWN	-3.5252

*MFS, major facilitator superfamily; GMC, glucose-methanol-choline; ABC, ATP-binding cassette; DOWN, down-regulated significantly; FALSE, no significant difference*.

## Conclusion

**FIGURE 6:**
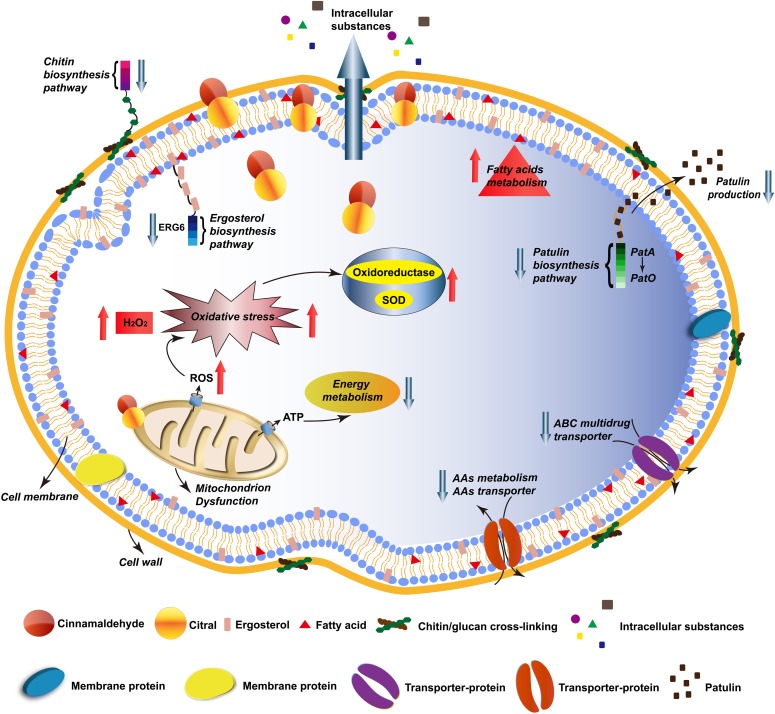
A schematic diagram illustrating the antifungal effects of Cin/Cit on *P. expansum* F-WY-12-02. AAs, amino acids; ABC, ATP-binding cassette.

As summarized in **Figure 6**, the lipophilic characters of EOs facilitated their access to the cytoplasmic membrane, then caused changes in membrane permeability, loss of intracellular substances, and wrinkles and depressions on cell surfaces. RNA-seq indicated that genes involved in amino sugar and nucleotide sugar metabolism (chitin biosynthesis), ergosterol biosynthesis, energy metabolism, AAs metabolism and ABC transporters (such as AAs transporter, ABC multidrug transporters) in *P. expansum* were mostly down-regulated, which probably affected the cellular primary structure and limited the nutrition transport, while reduced the drug resistance development. Particularly, PAT biosynthetic gene cluster was significantly down-regulated, directly causing lower levels of PAT production, which was verified by qRT-PCR and PAT measurement assays. Additionally, most genes associated with FAs metabolism were up-regulated, inducing more unsaturated FAs accumulation, which was a critical response mechanism of the cell to environmental stress. Besides, mitochondrial function and oxidation-reduction process were remarkably influenced, the dysfunction of MMP resulted in ROS accumulation, such as H_2_O_2_, then induced the activity of oxidoreductase. The physiological and biochemical character assays corresponded well with the RNA-seq result, which verified that Cin/Cit caused oxidative stress on *P. expansum*. These findings provide useful information to better understand the action mode of Cin/Cit on *P. expansum* at the molecular level and are conducive to develop more effective ways to prevent or control *P. expansum* contaminations.

## Ethics Statement

This article does not contain any studies with human participants or animals performed by any of the authors.

## Author Contributions

YW, YY, and TY conceived and designed the experiments. YW and KF performed the experiments. YW and KF analyzed the data. YW, ZZ, and HY drafted the manuscript. All authors read and approved the final manuscript.

## Conflict of Interest Statement

The authors declare that the research was conducted in the absence of any commercial or financial relationships that could be construed as a potential conflict of interest.
