# Supplementary material for: Effect of Cinnamaldehyde and Citral Combination on Transcriptional Profile, Growth, Oxidative Damage and Patulin Biosynthesis of Penicillium expansum
Source: Front Microbiol. 2018 Mar 29;9:597. doi: 10.3389/fmicb.2018.00597 (PMC5884930; doi:10.3389/fmicb.2018.00597)
Supplement: Supplementary file 1 [file Table_1.PDF]

SUPPLEMENTARY MATERIAL

Figure and Table Legends

Figure S1 Enzymatic disposal of superoxide and hydrogen peroxide. Superoxide is dismutated by SOD. Hydrogen peroxide is decomposed by CAT.

Table S1 MIC, MFC, $MIC_{cin/cit}$ and FICI of cinnamaldehyde and citral against *P. expansum* F-WY-12-02.

Figure S1

$$2O_2^{\bullet -} + 2H^+ \xrightarrow{\text{SOD}} H_2O_2 + O_2$$

$$2H_2O_2 \xrightarrow{\text{CAT}} 2H_2O + O_2$$

TABLE S1 MIC, MFC, $MIC_{cin/cit}$ and FICI of cinnamaldehyde and citral against *P. expansum* F-WY-12-02.

	MIC[a] (mg/L)	MFC[b] (mg/L)	$MIC_{cin/cit}$[c] (mg/L)	FICI[d]
cinnamaldehyde	90	100	45	1
citral	140	150	70	

[a] The minimum inhibitory concentration.

[b] The minimum fungicidal concentration.

[c] The minimum inhibitory concentration of cinnamaldehyde (Cin) and citral (Cit) in their combination (Cin/Cit), respectively.

[d] Fractional Inhibitory Concentration Index. Synergy (FICI ≤ 0.5), addition (0.5 < FICI ≤ 1), indifference (1 < FICI ≤ 4), or antagonism (FIC > 4).
